# Effects of Resistance Training and Soy Isoflavone on Body Composition in Postmenopausal Women

**DOI:** 10.1155/2010/156037

**Published:** 2010-05-18

**Authors:** Fábio Lera Orsatti, Eliana Aguiar Petri Nahas, Jorge Nahas-Neto, Nailza Maesta, Cláudio Lera Orsatti, Cesar Edurado Fernandes

**Affiliations:** ^1^Department of Gynecology and Obstetrics, Botucatu Medical School, Sao Paulo State University, Rubiao Junior, Botucatu, Sao Paulo 18618-970, Brazil; ^2^Department of Public Health, Center of Nutrition and Exercise Metabolism, Botucatu Medical School, Sao Paulo State University, Rubiao Junior, Sao Paulo 18618-970, Brazil; ^3^School of Physical Education, Federal University of Triangulo Mineiro (UFTM), Uberaba, Minas Gerais 38025-180, Brazil; ^4^Department of Gynecology and Obstetrics, ABC Medical School, Sao Paulo 09060-870, Brazil

## Abstract

*Objective*. To investigate the independent and additive effects of resistance training (RT) and soy isoflavone (ISO) on body composition in postmenopausal women (PW). *Method*. This study used a placebo-controlled, double-blind (soy), randomized (ISO versus placebo) × (RT versus No RT) design. A total of 80 PW, aged 45–70 years, were randomly (71 completed 9-months intervention): RT + ISO (*n* = 15), No RT + ISO (*n* = 20), RT + placebo (*n* = 18), and No RT + placebo (*n* = 18). ISO received 100 mg a day of isoflavone; and to RT attended supervised resistance training sessions. At baseline and 9-months, fat and muscle mass were estimated by DXA. ANOVA and test *t* were used. *Results*. RT groups showed significantly increased muscle strength (35.2%) and muscle mass (1.4%). Exercising attenuated gains in fat trunk and % body fat (*P* < .05). Significant decreases in muscle mass (−1.8%) and increases in fat mass of the whole-body (1.6%) and trunk (9.7%) was found in no-RT groups (*P* < .05). In ISO groups, there were no differences in body composition and muscle strength. ISO and RT had no additive effects. *Conclusion*. In PW: RT improved muscle mass and strength and attenuated gain of fat mass; ISO did not alter body composition and muscle strength; there were no additive effects of RT and ISO.

## 1. Introduction

A decrease in estrogen production in women at menopause is accompanied by changes in body composition which are characterized by body fat increase, primarily at the abdomen, and progressive reduction in muscle mass (sarcopenia) [1]. Sarcopenia is usually associated with functional impairment and physical disability, especially in women, and is the direct cause of reduction in muscle strength [2–4]. Menopause may induce a phase of rapid decreases in aerobic fitness, muscle strength, and balance, especially in sedentary women [5]. Several factors such as diet, lifestyle, as well as metabolic and hormonal parameters influence body composition in postmenopausal women [1, 6, 7]. 

The importance of physical activity at all stages of life for optimal bone health and muscle mass is established [8]. Aerobic exercise is important in maintaining overall health. Nonetheless, resistance muscle training may be more applicable to site-specific effects of exercise, besides having more favorable effects on maintaining or improving muscle mass and strength. Recently, we studied the effect of 16 weeks of resistance training (60%–80% of one-repetition maximum—1-RM) three times a week in 43 postmenopausal women, and we observed increases in maximal strength and muscle mass, indicating that resistance training may be applied in the rehabilitation or prevention of sarcopenia in postmenopausal women [9]. Besides, the abdominal fat reduction is associated with resistance training in older women [10, 11].

Isoflavones belong to a class of compounds called phytoestrogens. The primary isoflavones found in soy are genistein, daidzein, and glycitein. These nonsteroidal compounds are structurally [12] and weakly bound to estrogenic receptors [13]. Isoflavones may have a direct effect on muscle via estrogen receptors [14], increase of blood concentration of insulin-like growth factor-1 [15], or through their effects on reducing inflammation [16]. Besides, estrogen receptors are present in adipose tissue [12]. Thus, isoflavones could participate in the regulation of adiposity [12, 17]. 

However, the details of combined effects of regular resistance exercise and isoflavone on the prevention muscle loss or fat gain in women estrogen deficient remain to be solved. We observed that 16 weeks of supplementation with soy protein associated with resistance training did not result in greater increase in strength or muscle mass compared with placebo in postmenopausal women [10]. In this study, the time of intervention of the isoflavone could have affected the results. Two studies demonstrated association between muscle mass and isoflavone; however, the postmenopausal women received isoflavone for 24 weeks [18, 19]. 

Therefore, clinical trials of longer duration are needed to determine the effect of exercise training combined with isoflavone on body composition. Within this context, the primary purpose of this study was to determine the isolated and combined effects of resistance training and soy isoflavone on body composition (fat and muscle mass) in postmenopausal women.

## 2. Methods

### 2.1. Study Design and Participants

This clinical randomized, double-blind, placebo-controlled trial included 80 Brazilian women seen at the Climacterium Outpatient Service of the Department of Gynecology of Sao Paulo State University. All subjects were postmenopausal women aged 45–70 years with good overall health, sedentary, spontaneous amenorrhea for at least 12 months, and follicle-stimulating hormone level greater than 40 mIU/mL. Exclusion criteria included (1) high-fiber or high-soy diet, (2) hormone therapy (HT) or phytoestrogens use within the preceding 6 months, (3) noncontrolled high-blood pressure, (4) history of myopathic, neuropathic, and skeletal disorders, (5) history of breast cancer, endometrial carcinoma, cardiovascular disease, and thromboembolic disorders, (6) undiagnosed vaginal bleeding, (7) alcoholism, and (8) chronic gastrointestinal diseases. Subjects were considered to be sedentary when they reported no leisure physical activities besides everyday household tasks. Informed consent was obtained from all participants, and the study was approved by the Research Ethics Committee of Botucatu Medical School, UNESP.

Initial assessment consisted of case history taking, general and gynecological physical examination, oncotic colpocytology, mammography, and transvaginal ultrasonography. Data collected included information on age, menarche, time since menopause, parity, HT contraindication, weight, height, and waist circumference. After a prestudy period, participants were randomly assigned to one of two groups: ISO, receiving soy isoflavone extract (*n* = 40) or P, receiving placebo (*n* = 40). Examiners and subjects had no previous knowledge of group assignment (double-blind). Placebo and active treatment were identical in appearance. The centralized computerized subject randomization process was conducted by a statistician unaware of the study protocol using specific software. Capsule packages were labeled with code numbers and the statistician in charge was the only unblinded person.

Thus, 40 participants were given 250 mg of standardized soy extract (*Glycine max*), corresponding to 100 mg/day of isoflavone, twice a day in capsules containing 125 mg of soy extract plus 50 mg of isoflavones each, in glycoside form. The standardized extract contained approximately 50% of genistein and 35% of daidzein. The other 40 participants received two lactose capsules per day. All of the capsules were identical in appearance. Subjects were instructed to return any unused medication during visits for compliance determination. 

Subsequently, the participants were randomized to resistance training (RT) or No RT. Thus, 80 women were randomly assigned to one of four groups: RT + ISO (*n* = 20), No RT + ISO (*n* = 20), RT + P (*n* = 20), and No RT + P (*n* = 20). Followup length was nine months and evaluations were performed during the prestudy period, at baseline and at the end of the study. Nine women withdrew from the study (7 in RT and 2 in No RT) because of illness, family problems, or protocol violation (less than 2 weekly RT sessions). The remaining 71 subjects completed the nine-month study, and their data were included in the per-protocol analysis. 

All participants realized alimentary register. Three-day diaries (2 weekdays and 1 weekend day) and 24-hour recalls were completed at baseline of the study to estimate caloric intake and diet composition. Total energy, macronutrients (proteins, fats, and carbohydrates), and calcium contents were determined using “NutWin” (nutrition analysis software). Participants were advised to keep their usual dietary habits. The participants that showed inadequate data were oriented for ideal.

### 2.2. Exercise Intervention

The resistance-training protocol, as adapted for women over 45 years, consisted in the minimum 2 weekly sessions, on nonconsecutive days, for 9 months, under the supervision of trained personnel. Before training, subjects in the exercise group attended a 4-week adaptation period in order to get acquainted with the protocol. Initially, lighter loads were used and subjects performed one set of 15 repetitions at 40%–50% of 1-RM. Progression was gradual untill 3 sets of 8–12 repetitions at 60%–80% of 1-RM were performed [20]. The protocol consisted of dynamic exercises for both lower and upper limbs for a total of 50–60 minutes. Two exercises for each major muscle group (chest, back, and thigh) and one for each minor muscle group (biceps and triceps) were used, in 3 series of 8–12 repetitions. The load setting for each individual exercise was established by 1-RM testing. Loads were periodically adjusted at the end of each month to maintain loads at 8–12 repetitions maximum, according to strength gain and adaptation to training. Subjects performed one set of each of the following exercises: leg press, leg extension, peck deck, bench press, seated row, lat pull-down, triceps pulley, and biceps curl. Abdominal (3 sets of 30 repetitions) and calf exercises (3 sets of 20 repetitions with body weight) were also included. Air breathing was controlled by expiring during concentric actions and inspiring over eccentric actions in order to prevent apnea. Rest breaks between series and exercises ranged from 1 minute and 30 seconds to 2 minutes. During training sessions, participants were advised to perform an eccentric action in 2 second and a concentric action in 1 second. Subjects were considered as trained if they had worked out at least two times a week, on nonconsecutive days. Attendance was recorded by the trainers. Participants of the no-RT groups were asked to maintain their usual level of physical activity. No cardiovascular training was mentioned. 

Muscle strength was assessed in all subjects, by the 1-RM test, for dynamic exercises, after an adaptation period for strength testing (3–5 sessions on nonconsecutive days). The 1-RM was defined as the maximal weight that could be lifted with proper body alignment and the correct lifting technique. Muscle strength assessment included all of exercises at baseline, but only leg extension after nine months; because of cultural aspects related to strength, some women did not accomplish other tests. The 1-RM test was performed using the same apparatus used for resistance training.

### 2.3. Body Composition

Weight variation was assessed by the body mass index (BMI = weight/height^2^). Height and weight were obtained using a stadiometer (Seca, Brazil) and a standard balance beam scale (Filizola, Brazil), respectively, with subjects wearing lightweight clothing and no shoes. Weight was classified according to the system used by the World Health Organization (2000): 18.5 to 24.9 kg/m^2^ = normal weight, 25 to 29.9 kg/m^2^ = overweight, and ≥30.0 kg/m^2^ = obese [21]. Waist circumference was measured to the nearest 0.5 cm midway between the lowest rib margin and the iliac crest in supine position. Abdominal fat was indirectly assessed by measuring waist circumference, and was considered high when waist was >88 cm. 

Body composition (fat and lean mass) was assessed by dual-energy X-ray absorptiometry (DXA) at baseline and at nine months using a Hologic QDR-2000 densitometer plus scanner (Hologic, Waltham, MA, USA). To minimize interobserver variation, all scans and tests were carried out by the same investigator. Day-to-day percent coefficient of variations was <1.0% for whole-body. Whole body scans were divided into several regions such as arms, legs, trunk (pelvis, spine, and ribs), and head. Body composition was assessed by using the manual DXA analysis software (version 5.73A for whole body). The arm region was defined as the region extending from the head of the humerus to the distal tip of the fingers. The reference point between the head of the humerus and the scapula was positioned at the glenoid fossa. The leg region was defined as the region extending from the inferior border of the ischial tuberosity to the distal tip of the toes. The sub-whole-body was defined as the region extending from the shoulders to the distal tip of the toes. Reference points that could be clearly visualized on the DXA system terminal were selected.

### 2.4. Statistical Analysis

Statistical analyses were performed using the Software STATISTICA for Windows (version 6.0). The statistical distribution of the variables was evaluated by the tests of Shapiro-Wilk, Kolmogorov & Smirnov, and Levene. Normally distributed variables were reported as mean ± standard deviations. Differences among groups in baseline characteristics were evaluated using one-way analysis of variance. When differences were detected, a Tukey's post hoc test was performed to determine pairwise differences. Primary analyses included two-way repeated measures analysis of variance to test for interaction and for main effects of soy isoflavone and resistance training. All analyses were performed with no intention-to-treat. Comparison of daidzein and genistein levels between ISO and PL groups was carried out by an independent *t*-test. Exact *P* values were obtained from the tests employed. Statistical tests were two tailed and significance was set at 5%.

## 3. Results

Of the 80 postmenopausal women who were randomized, 71 completed this 9-month trial in the following four groups: RT + ISO (*n* = 15), No RT + ISO (*n* = 20), RT + P (*n* = 18), and No RT + P (*n* = 18). As expected, dropout rates were higher in the RT groups (*n* = 7) than in the No RT groups (*n* = 2) because of the time commitment. However, no differences in dropout rates were observed between ISO (*n* = 5) and Placebo (*n* = 4) groups.

At baseline, weight and BMI were lowers in the RT + P group compared with No RT + ISO and No RT + P, respectively, but not significantly different in other variables. Subject characteristics, fat, and muscle mass (MM) showed no significant differences among groups (Table 1). No significant differences were observed in energy or macronutrient intake per kg body mass (*P* > .05). Energy/kg intakes were 24 ± 6, 25 ± 7, 23 ± 6, and 22 ± 6 kcal/kg per day, carbohydrate/kg intakes were 2.5 ± 0.6, 2.2 ± 1.4, 2.2 ± 1.0, and 2.4 ± 0.6 g/kg/day, protein/kg intakes were 1.0 ± 0.4, 1.1 ± 0.5, 1.1 ± 0.6, and 1.1 ± 0.5 g/kg/day, and fat/kg intakes were 0.82 ± 0.32, 0.84 ± 0.29, 0.82 ± 0,34, and 0.83 ± 0.28 g/kg/day for RT + ISO, RT + P, No RT + ISO, and No RT + P, respectively.

**Figure 1:**
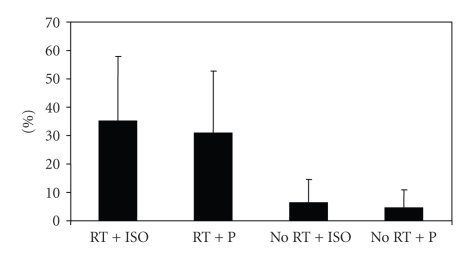
Percent changes in muscle strength (leg extension) from baseline to 9 months in response to exercise and isoflavone. ANOVA (p): ISO × Time = 0.82. RT × Time = 0.02. Interaction = 0.86.

Only groups with RT (RT + ISO and RT + P) increased the strength. However, no interaction between isoflavone and resistance training on muscle strength changes was found (Figure 1). There was a main effect of resistance training for muscle strength such that muscle fitness during the 9-month intervention was improved in the RT groups (35.2%) and reduced in the No RT groups (−1.1%). No main effect of soy isoflavone for muscle strength (ISO versus Placebo groups) was observed. 

Over body composition (fat and muscle mass), there was a main effect of resistance training (Table 2) such that MM improved in the RT groups (1.4% total-MM and 3.6% limb-MM), and decreased in the No RT groups (−1.8% total-MM and −0.7% limb-MM) during the 9-month intervention. Exercising attenuated fat trunk gains (RT = 6.1% versus No RT = 11.2%, *P* < .05) and % body fat (RT = 0.2% versus No RT = 2.1%, *P* < .05). No main effect of soy (ISO versus Placebo groups) was observed (Table 2). There were no significant interactions of isoflavone associated with resistance training on changes in fat mass or MM (Table 2). 

Table 3 shows that daidzein and genistein plasma concentrations significantly differed among groups after 9 months of intervention (*P* < .001). In the subjects given standardized soy extract, the detectable levels of both isoflavones were significantly higher than in the placebo group.

Seven of the 35 soy group subjects (20%) and four of the 36 placebo group subjects (11.1%) reported adverse experiences, most frequently in the gastrointestinal system. No serious adverse event related to isoflavone treatment was reported.

## 4. Discussion

The findings of this investigation assessing the isolated and combined effects of exercise and soy isoflavone for 9 months in postmenopausal women were the following: (1) resistance training favorably changed body composition increasing muscle mass and strength, and attenuating % body fat gains, (2) standardized soy extract (100 mg/day of isoflavone) did not change body composition (fat and muscle mass), and (3) there were no apparent additive or synergistic effects of soy isoflavone and resistance training on body composition. 

Important changes in the body composition of a female occur around the menopausal period. These changes include a decrease in bone, strength, and muscle mass and an increase in fat mass that can be managed by exercise, nutrition, and estrogen replacement, or by their combined intervention [1, 7, 8, 22]. In this study, the subjects engaged in resistance training gained strength and muscle mass (MM), whereas those in the nonexercising groups exhibited loss. Progressive resistance exercise or strength training is considered a promising intervention for reversing the loss of muscle function and the deterioration of muscle structure that is associate with aging [7, 23]. The American College of Sports Medicine currently recommends strength or resistance training as key components of an overall fitness program [20]. There is general consensus that physical exercise is a powerful intervention to obtain long-term benefits in muscle function, to reduce the frequency of falls, and to maintain independence and a high quality of life in older persons [3, 24]. Although the skeletal and muscular systems are structurally interdependent, both adapt to mechanical loading; however, the nature of their relationship is still unknown. The factors necessarily required to elicit changes to these systems have been studied. Fat-free has been suggested as a stronger determinant of bone mass with aging than either total mass or fat mass, although fat mass may also be an independent determinant [25]. 

In women, muscle mass and strength decline during perimenopause and this phenomenon seems to be estrogen dependent [1, 7]. Theoretically, estrogen has a direct anabolic action on the skeletal muscle that contains estrogenic receptors [14]. Aubertin-Leheudre et al. [18] investigated whether six months of isoflavone supplementation (70 mg of isoflavones/day) would increase fat-free mass and muscle mass index in obese-sarcopenic postmenopausal women. After 24 weeks, they found that limb and leg fat-free mass and muscle mass index (MMI = appendicular FFM/height^2^) significantly increased in the isoflavone group, but not in the placebo group. Although the data suggest that isoflavone improves muscle mass, we did not observe association in this study and other ones [10]. A possible explanation would be that these nonsteroidal compounds are weakly bound to estrogenic receptors (<1% of estradiol binding affinity) [13]. Besides, soy isoflavones preferentially bind to *β*-estrogen receptors that are found in the central nervous system, bones, vascular walls, and the urogenital tract. Unlike estrogens, isoflavones have little affinity with *α*-estrogen receptors [26], which are found in larger amount in the muscle [14]. Additionally, the form of isoflavones may impact the appropriateness of the dose used in present study. Izumi et al. [27] showed that the isoflavone aglycones were absorbed faster and in greater amounts than their glycosides in humans. Nevertheless, these findings have not been universal [28, 29]. 

 Though the weight and IMC have been smaller in RT + P, compared with other groups, the other variables of the corporal composition (muscle mass and body fat) and strength muscle did not show significant differences (Table 1). The postmenopausal women participating in our study were overweight, with an elevate rate of body fat and android fat distribution. Menopausal transition has been associated with a preferential increase in intraabdominal fat that is independent of age and total body fat mass [1], supporting the participation of sex hormones in body fat distribution. In this study, no reduction in body fat was observed after the nine-month intervention. Nonetheless, two weekly sessions of resistance training associated with lower trunk fat gains and lower % body fat gains. Resistance training has been shown to increase resting and total energy expenditure, and to induce decreases in total and abdominal fat [10, 11]. Hunter et al. [11] demonstrated that resistance training reduces the percentage of subcutaneous and intraabdominal fat in women, On the other hand, studies conducted in postmenopausal women showed no reduction in fat tissue [30, 31].

Little is known about the effects of isoflavones on body composition or fat and lean mass distribution in postmenopausal women. The rationale for studying the effect of soy protein on body fat distribution is that isoflavone may bind to estrogenic receptors of fat and lean tissues promoting gynoid fat deposition [19]. Goodman-Gruen and Kritz-Silverstein [32] investigated the association between dietary isoflavone intake and body composition in 208 participants to the Soy Health Effects Study (aged 45–74 years). They observed a significant inverse relation between soy consumption and body mass index, abdominal circumference and fat mass, as well as no correlation with lean mass, suggesting that an isoflavone-rich diet is associated with reduced body fat and may play a role in the prevention of obesity-related chronic diseases. 

In agreement with our findings, Moeller et al. [19] assessed body composition and physical activity in 69 perimenopausal women and found that 40 g of soy protein/day did not prevent abdominal fat deposition, but physical activity positively correlated with the regional distribution of fat and muscle mass. In 2007, Maesta et al. [10] assessed the effect of soy protein and progressive resistance training on body composition in postmenopausal women concluding that there were no additive effects of soy and exercise. Soy protein supplementation did not influence body composition indicators. Indeed, the increase in muscle mass and reduction in abdominal fat were correlated with resistance training. 

The findings herein reported should be interpreted with caution as this study presented some limitations. First, the sample size was relatively small due to the inclusion criteria and nature of the intervention (resistance training) used. Second, although the participants were oriented, the ingestion of food with isoflavone was not controlled. This might explain high daidzein and genistein plasma levels the in the placebo group, interfering in difference between perceptual changes. Finally, the dropout rate ranged from 2.5% in the No RT groups to 20% in the RT groups. These rates are high, but still comparable with those observed in other trials involving exercising [33]. As a matter of fact, the most frequently reported reason for dropping-out and poor compliance was lack of interest to continue with the resistance-training protocol. This study was designed to assess the effect of resistance training combined with soy isoflavone on body composition in the women who completed the program, and not to determine the effectiveness of such intervention in a public health or community setting. Thus, the subjects who dropped out or did not meet compliance requirements were not retested and no intention-to-treat analysis was performed post hoc.

In conclusion, our results indicate that standardized soy extract (100 mg/day of isoflavone) did not change body composition (fat and muscle mass) and that soy isoflavone and resistance training had no apparent additive or synergistic effects. Resistance training was found to be a safe and effective intervention for reversing the loss of muscle function by increasing muscle mass and strength and to attenuate fat trunk gains and % body fat in postmenopausal women.

## Figures and Tables

**Table 1:** Baseline clinical characteristics of 71 postmenopausal women (mean ± SD).

Variables	RT + ISO	RT + P	No RT + ISO	No RT + P	*P* value*
	(*n* = 15)	(*n* = 18)	(*n* = 20)	(*n* = 18)	
Age (years)	55.7 ± 7.0	56.6 ± 8.8	56.0 ± 5.8	55.3 ± 8.0	.99
Postmenopause (years)	7.7 ± 5.3	8.7 ± 6.1	6.5 ± 4.9	7.7 ± 6.2	.64
FSH (mUI/mL)	74.0 ± 27.7	71.8 ± 24.3	63.0 ± 21.1	63.3 ± 21.3	.15
E_2_ (pg/mL)	23.1 ± 4.8	21.7 ± 4.3	22.5 ± 4.8	23.3 ± 5.8	.59
Weight (kg)	73.2 ± 15.1	62.8 ± 9.8^(a)^	75.0 ± 12.9^(a)^	73.7 ± 12.6	.03
Height (cm)	154.9 ± 7.0	154.7 ± 5.6	158.1 ± 5.8	155.6 ± 5.2	.27
WC (cm)	95.7 ± 10.1	87.3 ± 7.7	94.1 ± 12.3	95.1 ± 11.9	.09
BMI (kg/m^2^)	30.3 ± 4.7	26.0 ± 3.0^(a)^	30.1 ± 4.7	30.4 ± 5.3^(a)^	.03
MM (kg)	34.0 ± 6.2	30.3 ± 4.1	34.0 ± 4.2	33.6 ± 4.0	.08
MM-Limbs (kg)	13.6 ± 2.8	12.0 ± 1.9	13.7 ± 2.0	13.6 ± 2.0	.08
MM (%)	52.2 ± 4.5	54.3 ± 4.2	51.2 ± 6.2	51.0 ± 5.1	.20
Trunk fat mass (kg)	14.3 ± 4.4	12.3 ± 3.2	15.8 ± 5.4	14.8 ± 4.4	.11
Body fat (%)	46.2 ± 4.9	43.0 ± 4.4	46.8 ± 6.7	46.5 ± 5.7	.12
Strength muscle (kg)	30.7 ± 9.2	31.3 ± 6.8	33.0 ± 10.2	32.7 ± 7.9	.15

FSH: follicle-stimulating hormone; E_2_: estradiol; WC: waist circumference; BMI: body mass index; MM: muscle mass; SD: standard deviations; ISO: isoflavone; PL: placebo; RT: resistance training.

*Significant difference among groups (*P* < .05) (one-way ANOVA),^(a)^With significantly difference between groups (*P* < .05).

**Table 2:** Effects of soy (ISO) and/or exercise (RT) on body composition (fat and muscle mass) in the study groups at baseline and at 9 months (mean ± SD).

Variables	RT + ISO	RT + P	No RT + ISO	No RT + P
	(*n* = 15)	(*n* = 18)	(*n* = 20)	(*n* = 18)
MM (Kg)				
Baseline	34.0 ± 6.2	30.3 ± 4.1	34.0 ± 4.2	33.6 ± 4.0
After 9 months	34.6 ± 6.1	30.6 ± 3.7	33.2 ± 3.8	33.1 ± 4.1
ANOVA (p)				
ISO × Time	0.61			
RT × Time	<0.01			
Interaction	0.19			
Limb-MM (kg)				
Baseline	13.6 ± 2.8	12.0 ± 1.9	13.7 ± 2.0	13.6 ± 2.0
After 9 months	14.2 ± 2.9	12.3 ± 1.9	13.7 ± 2.2	13.4 ± 1.9
ANOVA (p)				
ISO × Time	0.05			
RT × Time	<0.00			
Interaction	0.67			
MM (%)				
Baseline	52.2 ± 4.5	54.3 ± 4.2	51.2 ± 6.2	51.0 ± 5.1
After 9 months	52.4 ± 4.8	54.3 ± 5.0	50.0 ± 6.0	50.3 ± 5.6
ANOVA (p)				
ISO × Time	0.79			
RT × Time	0.04			
Interaction	0.48			
Trunk fat mass (kg)				
Baseline	14.3 ± 4.4	12.3 ± 3.2	15.8 ± 5.4	14.8 ± 4.4
After 9 months	15.4 ± 5.3	12.9 ± 3.7	17.2 ± 6.1	16.7 ± 5.6
ANOVA (p)				
ISO × Time	0.96			
RT × Time	0.04			
Interaction	0.28			
Whole-body fat mass (kg)				
Baseline	31.1 ± 10.3	24.8 ± 6.2	32.2 ± 9.7	31.3 ± 8.8
After 9 months	31.8 ± 10.0	25.6 ± 7.4	33.8 ± 10.7	32.8 ± 9.2
ANOVA (p)				
ISO × Time	0.94			
RT × Time	0.12			
Interaction	0.85			
Body fat (%)				
Baseline	46.2 ± 4.9	43.0 ± 4.4	46.8 ± 6.7	46.5 ± 5.7
After 9 months	45.6 ± 5.1	43.8 ± 5.8	48.4 ± 6.8	48.0 ± 6.1
ANOVA (p)				
ISO × Time	0.25			
RT × Time	0.02			
Interaction	0.20			

**Table 3:** Daidzein and genistein plasma levels at 9 months of intervention (mean ± SD).

Parameter	Isoflavone	Placebo	*P*-values*
	(*n* = 35)	(*n* = 36)	
Daidzein (*μ*mol/dL)	220.4 ± 53.5	125.4 ± 27.9	<.0001
Genistein (*μ*mol/dL)	144.3 ± 50.5	68.1 ± 19.5	<.0001

*Significant difference among groups (*P* < .05) (independent *t*-test).

## References

[1] Sowers M, Zheng H, Tomey K (2007). Changes in body composition in women over six years at midlife: ovarian and chronological aging. *Journal of Clinical Endocrinology and Metabolism*.

[2] Evans WJ (2002). Effects of exercise on senescent muscle. *Clinical Orthopaedics and Related Research*.

[3] Janssen I, Heymsfield SB, Ross R (2002). Low relative skeletal muscle mass (sarcopenia) in older persons is associated with functional impairment and physical disability. *Journal of the American Geriatrics Society*.

[4] Lebrun CE, van der Schouw YT, de Jong FH, Grobbee DE, Lamberts SW (2006). Fat mass rather than muscle strength is the major determinant of physical function and disability in postmenopausal women younger than 75 years of age. *Menopause*.

[5] Asikainen TM, Kukkonen-Harjula K, Miilunpalo S (2004). Exercise for health for early postmenopausal women: a systematic review of randomised controlled trials. *Sports Medicine*.

[6] Toth MJ, Tchernof A, Sites CK, Poehlman ET (2000). Effect of menopausal status on body composition and abdominal fat distribution. *International Journal of Obesity*.

[7] Sirola J, Rikkonen T (2005). Muscle performance after the menopause. *Journal of the British Menopause Society*.

[8] Kohrt WM, Bloomfield SA, Little KD, Nelson ME, Yingling VR (2004). American College of Sports Medicine Position Stand: physical activity and bone health. *Medicine and Science in Sports and Exercise*.

[9] Orsatti FL, Nahas EA, Maesta N, Nahas-Neto J, Burini RC (2008). Plasma hormones, muscle mass and strength in resistance-trained postmenopausal women. *Maturitas*.

[10] Maesta N, Nahas EA, Nahas-Neto J (2007). Effects of soy protein and resistance exercise on body composition and blood lipids in postmenopausal women. *Maturitas*.

[11] Hunter GR, Bryan DR, Wetzstein CJ, Zuckerman PA, Bamman MM (2002). Resistance training and intra-abdominal adipose tissue in older men and women. *Medicine and Science in Sports and Exercise*.

[12] Anwar A, McTernan PG, Anderson LA (2001). Site-specific regulation of oestrogen receptor-*α* and -*β* byoeatradiol in human adipose tissue. *Diabetes, Obesity and Metabolism*.

[13] Mackey R, Eden J (1998). Phytoestrogens and the menopause. *Climacteric*.

[14] Lemoine S, Granier P, Tiffoche C, Rannou-Bekono F, Thieulant ML, Delamarche P (2003). Estrogen receptor alpha mRNA in human skeletal muscles. *Medicine and Science in Sports and Exercise*.

[15] Arjmandi BH, Lucas EA, Khalil DA (2005). One year soy protein supplementation has positive effects on bone formation markers but not bone density in postmenopausal women. *Nutrition Journal*.

[16] Chilibeck PD, Cornish SM (2008). Effect of estrogenic compounds (estrogen or phytoestrogens) combined with exercise on bone and muscle mass in older individuals. *Applied Physiology, Nutrition and Metabolism*.

[17] Sites CK, Cooper BC, Toth MJ, Gastaldelli A, Arabshahi A, Barnes S (2007). Effect of a daily supplement of soy protein on body composition and insulin secretion in postmenopausal women. *Fertility and Sterility*.

[18] Aubertin-Leheudre M, Lord C, Khalil A, Dionne IJ (2007). Six months of isoflavone supplement increases fat-free mass in obese-sarcopenic postmenopausal women: a randomized double-blind controlled trial. *European Journal of Clinical Nutrition*.

[19] Moeller LE, Peterson CT, Hanson KB (2003). Isoflavone-rich soy protein prevents loss of hip lean mass but does not prevent the shift in regional fat distribution in perimenopausal women. *Menopause*.

[20] Kraemer WJ, Adams K, Cafarelli E (2002). American College of Sports Medicine position stand. Progression models in resistance training for healthy adults. *Medicine and Science in Sports and Exercise*.

[21] WHO (2000). *Obesity: Preventing and Managing the Global Epidemic. Report of a WHO Consultation*.

[22] NAMES (2006). Management of osteoporosis in postmenopausal women: 2006 position statement of The North American Menopause Society. *Menopause*.

[23] Evans EM, Racette SB, Van Pelt RE, Peterson LR, Villareal DT (2007). Effects of soy protein isolate and moderate exercise on bone turnover and bone mineral density in postmenopausal women. *Menopause*.

[24] Asbury EA, Chandrruangphen P, Collins P (2006). The importance of continued exercise participation in quality of life and psychological well-being in previously inactive postmenopausal women: a pilot study. *Menopause*.

[25] Gjesdal CG, Halse JI, Eide GE, Brun JG, Tell GS (2008). Impact of lean mass and fat mass on bone mineral density: the Hordaland Health Study. *Maturitas*.

[26] Morito K, Hirose T, Kinjo J (2001). Interaction of phytoestrogens with estrogen receptors *α* and *β*. *Biological and Pharmaceutical Bulletin*.

[27] Izumi T, Piskula MK, Osawa S (2000). Soy isoflavone aglycones are absorbed faster and in higher amounts than their glucosides in humans. *Journal of Nutrition*.

[28] Setchell KD, Brown NM, Desai P (2001). Bioavailability of pure isoflavones in healthy humans and analysis of commercial soy isoflavone supplements. *Journal of Nutrition*.

[29] Zubik L, Meydani M (2003). Bioavailability of soybean isoflavones from aglycone and glucoside forms in American women. *American Journal of Clinical Nutrition*.

[30] Elliott KJ, Sale C, Cable NT (2002). Effects of resistance training and detraining on muscle strength and blood lipid profiles in postmenopausal women. *British Journal of Sports Medicine*.

[31] Teixeira PJ, Going SB, Houtkooper LB (2003). Resistance training in postmenopausal women with and without hormone therapy. *Medicine and Science in Sports and Exercise*.

[32] Goodman-Gruen D, Kritz-Silverstein D (2003). Usual dietary isoflavone intake and body composition in postmenopausal women. *Menopause*.

[33] Bonaiuti D, Shea B, Iovine R (2002). Exercise for preventing and treating osteoporosis in postmenopausal women. *Cochrane Database of Systematic Reviews*.

